# RIP sequencing in mantle cell lymphoma identifies functional long non-coding RNAs associated with translation machinery

**DOI:** 10.1038/s41408-019-0216-6

**Published:** 2019-07-26

**Authors:** Guangzhen Hu, Yuji Zhang, Mamta Gupta

**Affiliations:** 1 Division of Hematology, Mayo Clinic, Rochester, MN 55905 USA; 2 Department of Health Sciences, Mayo Clinic, Rochester, MN 55905 USA; 3 Department of Biochemistry and Molecular Medicine, School of Medicine and Health Sciences, The George Washington University, GW Cancer Center, Washington, DC USA

**Keywords:** Cancer, Cell signalling

## To the Editor

Mantle cell lymphoma (MCL) is a type of aggressive non-Hodgkin B cell lymphoma derived from naive pre-germinal center cells of mantle zones^1^. Despite advances in the development of targeted therapy, prognosis of MCL is poor, with a median survival of 5–7 years^2^. The pathogenesis of MCL is complex due to molecular alterations at several regulatory hubs. Regulatory control of gene transcription is well studied in most of the cancers, including MCL; however, the regulatory mechanisms of protein translation in cancers including MCL are less understood. Translation machinery, also referred as translation initiation complex (eIF4F), regulates the translation of mRNAs mainly via translation initiation factor-4E (eIF4E). We and others have previously shown that eIF4E is overexpressed in MCL tissue, and is correlated with poor prognosis^3,4^.

Long non-coding RNAs (lncRNAs) are vaguely defined by the length range of >200 nucleotides to several kilobases of RNA, and they lack coding potential. Due to their important role in the epigenetic process of gene expression lncRNAs have recently received considerable attention. While transcriptional role of lncRNAs in cancers is well studied^5^, the role of lncRNAs in translation regulation has been revealed only in recent years. We have recently reported that lncRNA ROR-AS1 is highly upregulated in MCL tissue and regulates gene transcription via the epigenetic repressive complex^6^. Recent ribosome profiling as well as polysome profiling approaches have presented evidence suggesting that some lncRNAs can be found associated with ribosomal and poly-ribosomal fractions^7^. This raises the possibility that ribosome-bound lncRNAs can be involved in fine-tuning the speed or specificity of the translation machinery.

**Fig. 1:**
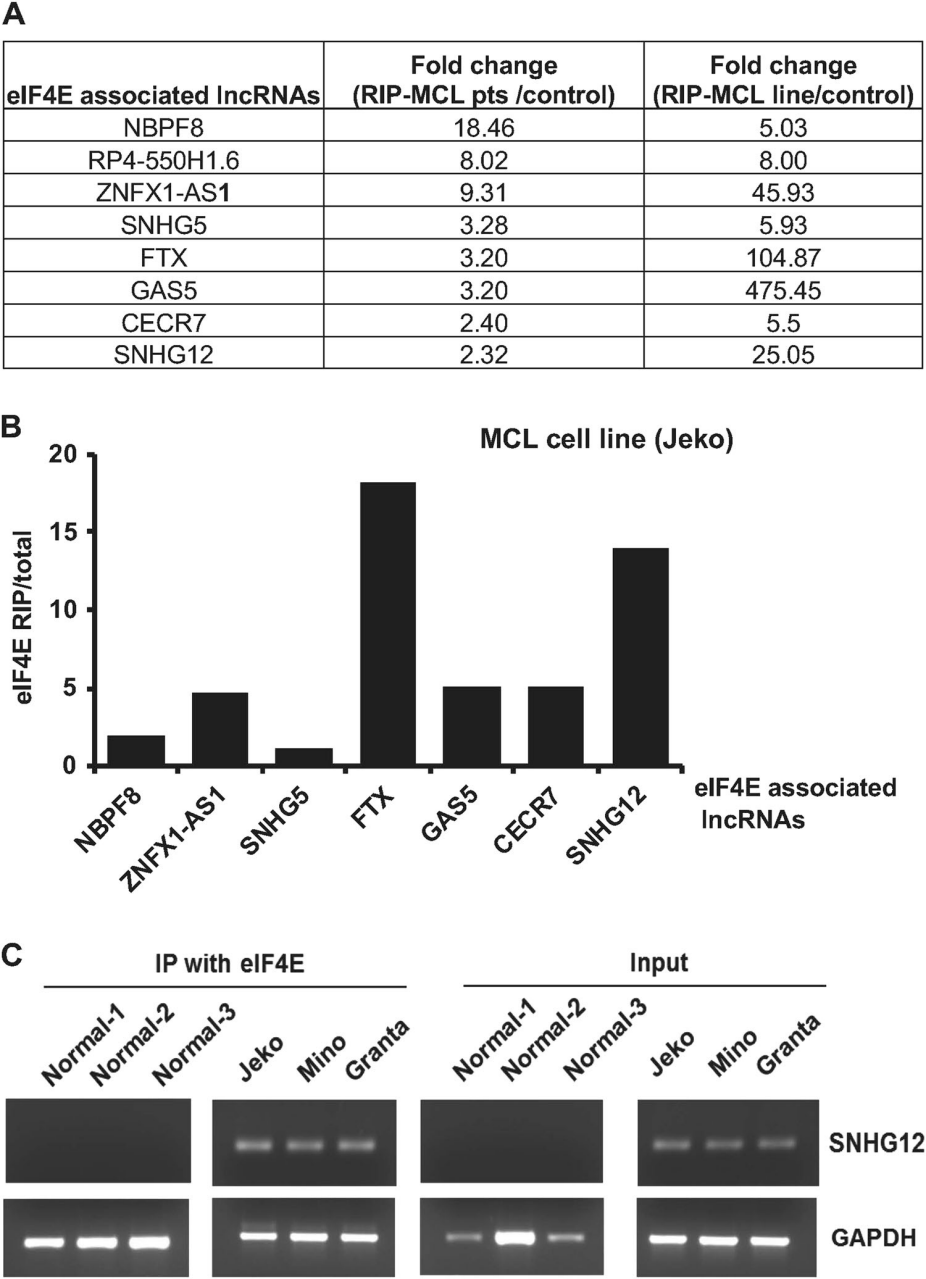
RNA sequencing demonstrating eIF4E-associated lncRNAs in tumor cells from MCL patients and MCL cell line. **a** Fold change of top eight lncRNAs detected in RNA-IP using anti-eIF4E antibody in MCL tumor cells (*n* = 4) and Jeko cell line as compared to normal control (*n* = 3) are shown. **b** Enrichment analysis on the lncRNAs by comparing the mapped read counts in the RNA sample pulled down with ant-eIF4E antibody to total RNA samples in Jeko cell line. **c** SNHG12 was detected by RT-PCR after RNA-IP assay using eIF4E antibody in MCL cell lines (*n* = 3) Jeko, Mino, and Granta and normal control (*n* = 3). GAPDH was used as a control

To identify the translation machinery-associated lncRNAs in MCL, we used tumor cells from MCL patients and corresponding normal lymph node controls and performed RNA-immunoprecipitation (RIP)-sequencing. RNA extracted from MCL patients (*n* = 4), MCL cell line (Jeko), and normal controls (*n* = 3) were pulled down with anti-eIF4E antibody, followed by RNA sequencing and secondary analysis to detect lncRNAs. Specific binding of lncRNAs with eIF4E was evaluated by enrichment analysis, in which the reading counts mapped to the genes of the RNA sample immunoprecipitated with eIF4E (IP) were compared with those of the total RNA sample. Data analysis demonstrated hundreds of lncRNAs associated with eIF4E. Based on twofold changes, we selected the top eight eIF4E-enriched lncRNAs in the MCL patient samples, including novel (NBPF8 and RP4-550H1.6) and known-lncRNAs (ZNFX1-AS1, SNHG5, FTX, GAS5, CECR7, and SNHG12) as compared to normal controls (Fig. 1a and S1). We next confirmed these eight lncRNAs in the eIF4E RIP-sequencing data in Jeko MCL cell line by differential binding analysis. Results confirmed that these lncRNAs are indeed associated with eIF4E in the Jeko cells, although the expression level varied between MCL patient and MCL cell lines (Fig. 1a). Further enrichment analysis in the Jeko showed that FTX and SNHG12 had more than 15-fold higher enrichment with ant-eIF4E antibody,indicating specific binding of the lncRNAs with eIF4E **(**Fig. 1b**)**. To further confirm direct association of SNHG12 lncRNA with eIF4E, we performed RNA-IP in three MCL lines (Jeko, Mino, and Granta) and in normal controls (*n* = 3) with IgG and eIF4E antibodies, followed by targeted reverse transcriptase (RT)-PCR. RT-PCR data showed that SNHG12 was highly enriched after RNA-IP with anti-eIF4E antibody as compared to IgG control in all three MCL cell lines tested, while normal B cells had no demonstrable SNHG12 enrichment with anti-eIF4E IP (Fig. 1c). Overall these data suggest that some lncRNAs in fact are associated with a translation machinery in MCL and might play a role in translation regulation.

**Fig. 2:**
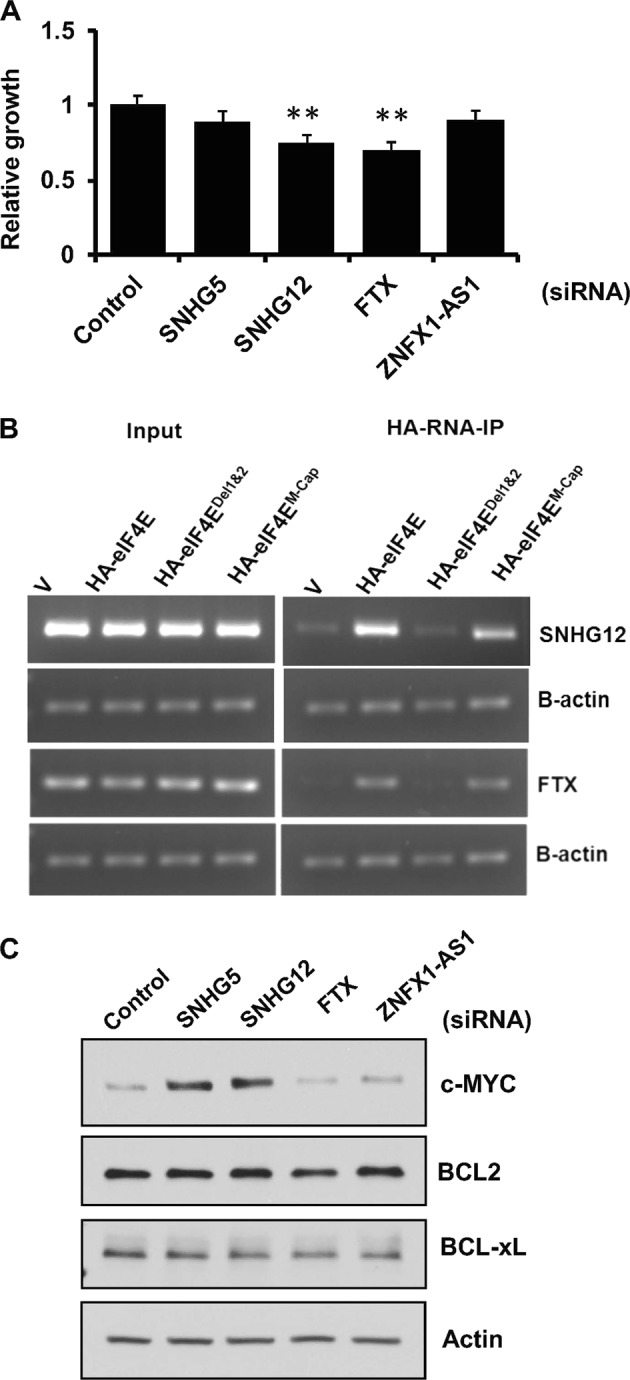
Functional studies of eIF4E-associated lncRNAs. **a** Bar graphs showing the effect of lncRNAs SNHG5, SNHG12, FTX, and ZNFX1 on growth of cells. The specified lncRNAs were knocked down by transfection of targeted siRNA, and 48 h after transfection cells were plated in 96-well plates for growth assessment by MTT assay. Bars represent mean ± SD from eight replicates; **p < 0.001. **b** Images of agarose gels showing total levels (left panel) and eIF4E-bound (right panel) lncRNAs SNHG12 and FTX in 293T cells transfected with empty vector (V), HA-eIF4E^WT^, HA-eIF4E^Del1&2^, or HA-eIF4E^M-cap^ lncRNA levels were detected by RT-PCR using total RNA (left) or RNA-IP with anti-HA antibody. β-Actin was used as a loading control. **c** Images of western blots showing protein levels of c-MYC, BCL-2, and BCL-XL in cells transfected with a control or lncRNA targeted siRNA to individually knock down each lncRNA

Furthermore, siRNA-mediated inhibition of SNHG12 or FTX (but not SNHG5 or ZNFX1-AS1) reduced the 20–25% proliferation as assessed by the MTT assay (Fig. 2a; S2A–D). FTX and SNHG12 lncRNAs have been previously shown to play important roles in other cancers^8,9^, but their roles in protein translation or MCL growth are not known. It has been known that eIF4E uses cap-binding motif to bind mRNAs to be translated^10^, while we have previously shown that eIF4E uses RNA-binding motifs to interact with lncRNA GAS5 (ref. ^7^). To examine the relative importance of RNA-binding and cap binding motifs of eIF4E for the interaction between eIF4E and lncRNAs, various plasmid constructs: (i) eIF4E (HA-eIF4E^WT^), (ii) eIF4E with RNA-binding motifs deleted (HA-eIF4E^Del1&2)^, and (iii) cap mutant (HA-eIF4E^cap mutant^), were transfected into HEK-293T cells and RNA-IP was performed for the lncRNAs FTX and SNHG12. There was a net 3–5-fold increase in binding of SNHG12 or FTX lncRNAs in cells overexpressing HA-eIF4E^WT^ as compared to those transfected with an empty vector. However, overexpression of HA-eIF4E^Del1&2^ but not the HA-eIF4E^cap mutant^ decreased the binding between eIF4E and SNHG12 or FTX lncRNAs (Fig. 2b). We then tested whether these lncRNAs (SNHG5, SNGH12, FTX, and ZNFX1-AS1) are able to regulate translation of the eIF4E downstream target genes *c-Myc, BCL2*, and *Bcl-xl*. As shown in Fig. 2c, MYC but not BCL2 or BCL-XL protein levels were markedly increased after siRNA-mediated knockdown of SNHG5 or SNHG12; also, the global protein translation was not affected (Fig. 2c; S3A). Moreover, the MYC mRNA level remained unaffected at least with knockdown of SNHG12 lncRNA, although a modest decrease in MYC mRNA was observed with knockdown of SNHG5, suggesting that SNHG12 lncRNA can modulate MYC expression at a translation level (S3B).

In summary, to the best of our knowledge, we report for the first time that several lncRNAs including SNHG12, FTX, SNHG5, and ZNFX1-AS1 are found to be associated with translation machinery via eIF4E in MCL tumor cells. We also demonstrate that RNA-binding motifs (but not cap-binding domain) of eIF4E are responsible for binding of lncRNAs with eIF4E. Among lncRNAs that bound to eIF4E, at least SNHG5 and SNHG12 can modulate c-Myc translation in MCL cells. Future work will need to clarify whether eIF4E-bound or ribosome-bound lncRNAs are capable of eliciting additional regulatory roles to the translation machinery. Our results expand the known repertoire of translation regulation and lncRNA biology for potential implications in cancer therapies. In future studies may clarify if eIF4E- or ribosome-bound lncRNAs elicite additional regulatory roles on translation machinery.

## Supplementary information

S1

S2

S3
