# Supplementary figures and images for: RIP sequencing in mantle cell lymphoma identifies functional long non-coding RNAs associated with translation machinery
Source: Blood Cancer J. 2019 Jul 26;9(8):55. doi: 10.1038/s41408-019-0216-6 (PMC6659685; doi:10.1038/s41408-019-0216-6)

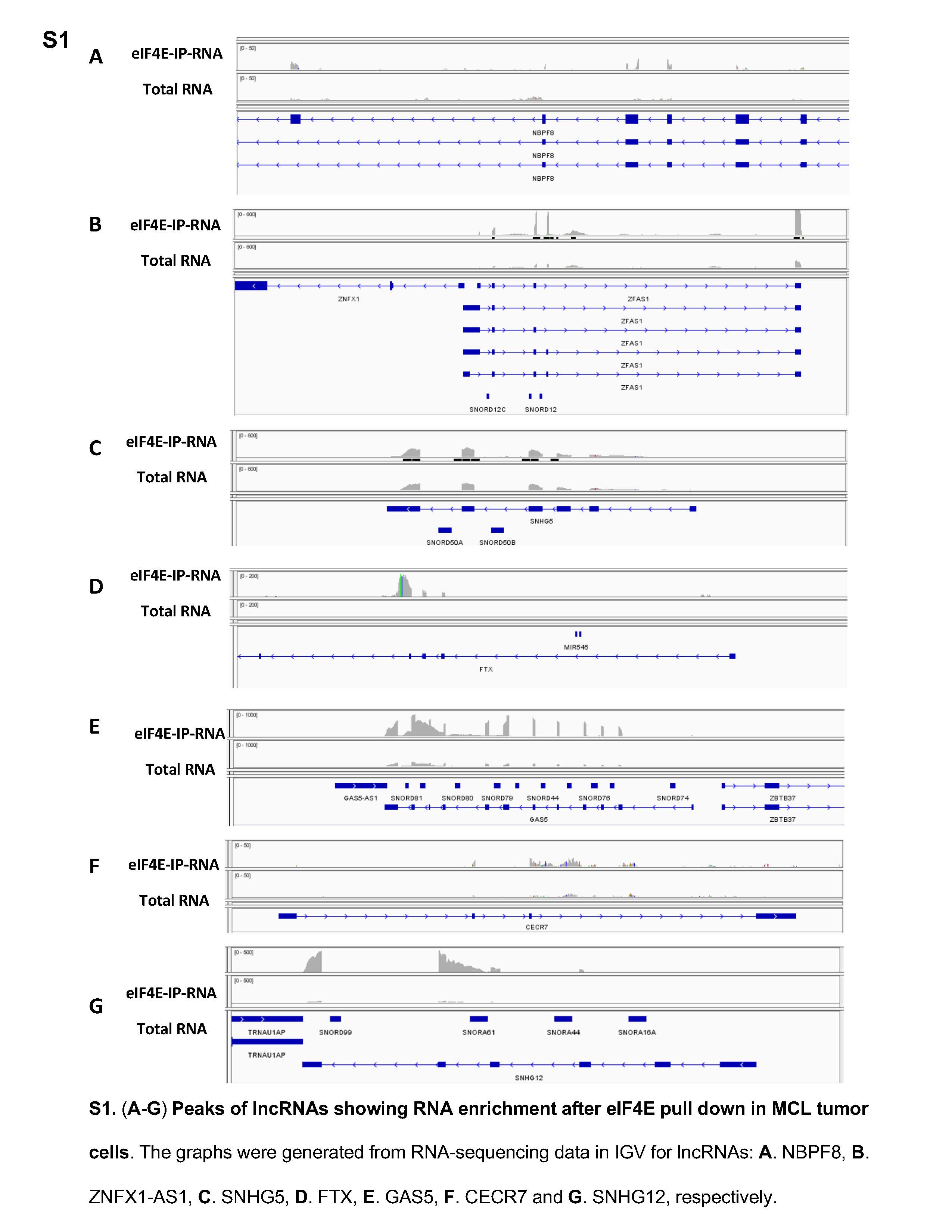

Supplement: Supplementary file 1 — S1 [file 41408_2019_216_MOESM1_ESM.jpg]

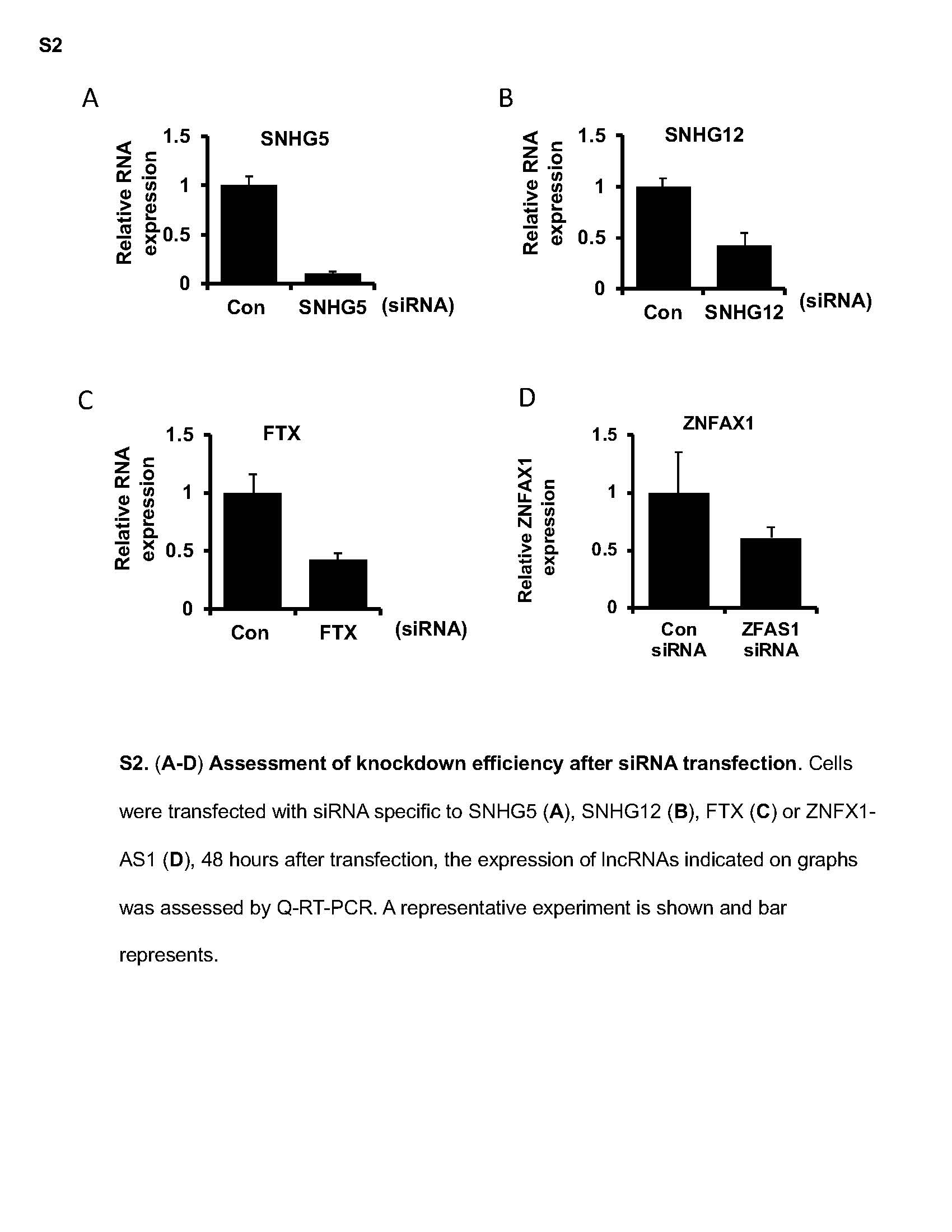

Supplement: Supplementary file 2 — S2 [file 41408_2019_216_MOESM2_ESM.jpg]

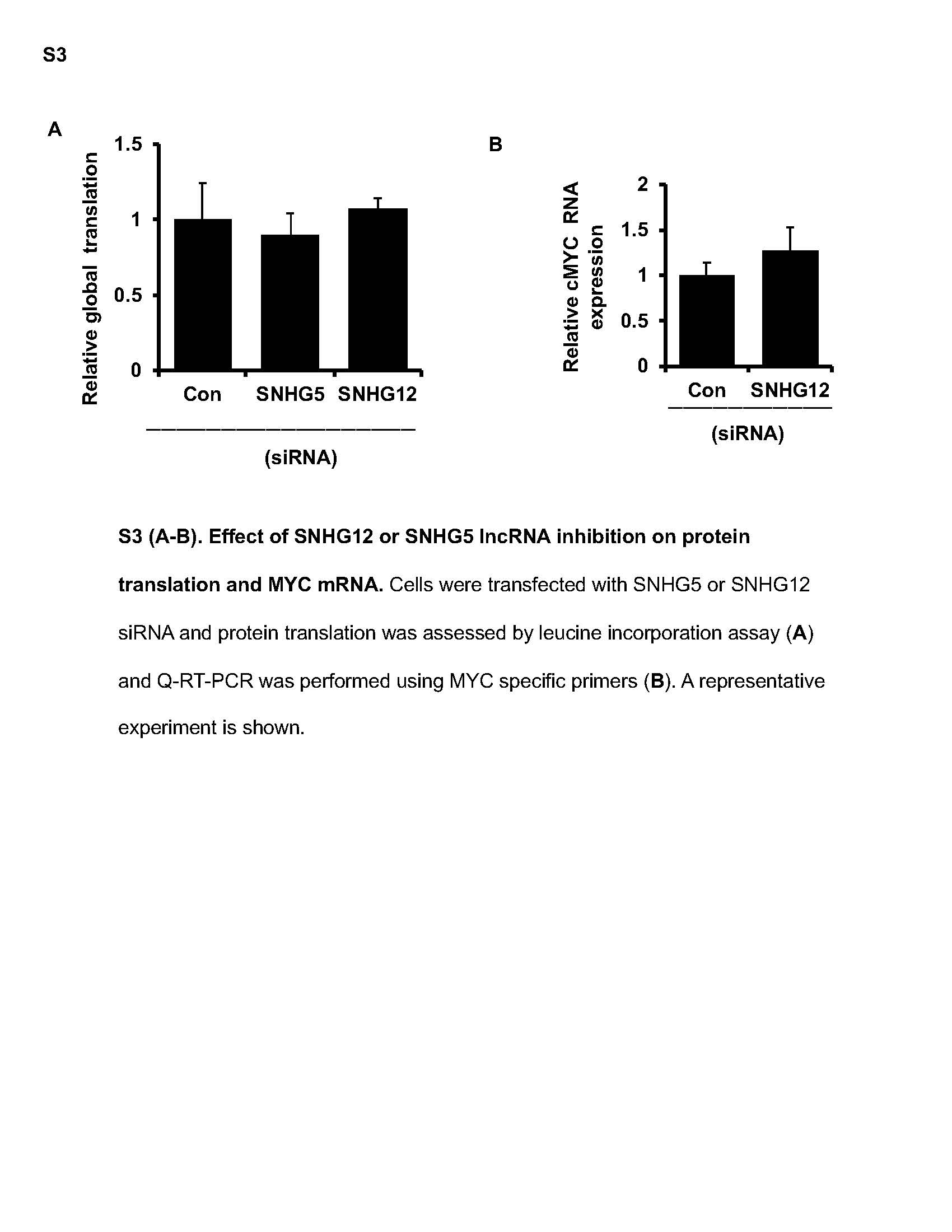

Supplement: Supplementary file 3 — S3 [file 41408_2019_216_MOESM3_ESM.jpg]
